# The Management of Wound Healing in Infections after Hip Arthoplasty Using Stimulan and Negative Pressure Wound Therapy

**DOI:** 10.3390/diagnostics14192206

**Published:** 2024-10-03

**Authors:** Florentin Dimofte, Cristina Dimofte, Sorin Ungurianu, Cristina Serban, George Țocu, Nicoleta Cârneciu, Iulia Filip, Laura Bezman, Ana Fulga, Dana Tutunaru, Irina Mihaela Abdulan, Bogdan Mihnea Ciuntu, Raul Mihailov, Alin Mihai Vasilescu, Dorel Firescu

**Affiliations:** 1 General Surgery Clinic, “St. Apostol Andrei” County Emergency Clinical Hospital, 800578 Galați, Romania; florentin.dimofte@ugal.ro (F.D.); cristina.serban@ugal.ro (C.S.); george.tocu@ugal.ro (G.Ț.); nicoleta.carneciu@ugal.ro (N.C.); iulia.filip@ugal.ro (I.F.); 2 Department of Morphological and Functional Sciences, Faculty of Medicine “Dunarea de Jos”, 800008 Galați, Romania; sorinungurianu@yahoo.com (S.U.); ana.fulgaa@gmail.com (A.F.); 3 Department Radiology, “Saint John” Emergency Children Hospital, Str. Gheorghe Asachi, Nr.2, 800487 Galați, Romania; cristina.dimofte90@yahoo.com; 4 Department of General Surgery, Faculty of Medicine “Dunarea de Jos”, 800008 Galati, Romania; raul.mihailov@ugal.ro (R.M.); dorelfirescu@yahoo.com (D.F.); 5 Department of Laboratory Medicine, Faculty of Medicine “Dunarea de Jos”, 800008 Galați, Romania; dana_tutunaru_cmgl@yahoo.com; 6 Department of Ophtalmology, Faculty of Medicine “Dunarea de Jos”, 800008 Galati, Romania; laura.bezman@ugal.ro; 7 Department of Medical Specialties I, “Grigore T. Popa” University of Medicine and Pharmacy, 700115 Iași, Romania; 8 Department of General Surgery, “Grigore T. Popa” University of Medicine and Pharmacy, 700115 Iași, Romania; alin.vasilescu@umfiasi.ro

**Keywords:** hip arthroplasty, infection, biocomposite, negative pressure wound therapy

## Abstract

Background: medical teams continue to face challenges with infections following hip replacement surgery, whether they occur shortly after the procedure or months or years later. Certain medical conditions like diabetes, rheumatoid arthritis, and obesity are risk factors that make patients more susceptible to infections. Traditional intervention methods such as DAIR, one-step, or two-step procedures are being enhanced and refined to ensure quicker and more effective treatment. Some cases present particularly difficult challenges, featuring persistent fistulas and unpredictable responses to treatment. Methods: in our article, we share two unique cases, detailing their histories, progressions, and treatment decisions. We explore the use of antibiotic-impregnated calcium biocomposite as a local adjuvant therapy and the application of negative pressure therapy to expedite healing. The system of NWPT has seen widespread uptake and is now implemented routinely for open wounds, such as open fractures, fasciotomies, ulcers, and infected wounds. Results: our findings demonstrate that surgical debridement and calcium sulfate bead insertion successfully treat bone and joint infections without causing any side effects or complications. As a particularity, in the first case, we encountered the exteriorization of Stimulan pearls after surgery, without other complications related to the biocomposite. Conclusions: we have found that NPWT is a beneficial tool in managing complex wounds in both acute and chronic stages, after the infection is cured, reducing the need for frequent dressing changes, shortening hospital stays, and enhancing patient comfort.

## 1. Introduction

Infections following total hip arthroplasty (THA) can pose challenging complications, affecting both the financial aspects of the healthcare system and patient well-being [1]. The occurrence of prosthetic joint infection (PJI) after primary THA is approximately 1–2%.

Various categories of risk factors have been identified in the literature for the development of PJI: pathologies such as diabetes, urinary tract infections, septic arthritis, dental issues, anemia, psychiatric disorders, osteoporosis, and hypothyroidism. Protective factors include the use of statins, preoperative decolonization, and preadmission skin preparation [2].

Additionally, factors such as blood transfusion, prolonged operative time, and previous surgery are frequently identified among the surgery/hospital-associated risk factors [3]. As the population ages, these risk factors become more prevalent, and as a result, the rates of complications after THA are expected to rise.

Coagulase-negative Staphylococci are found in approximately 30–41% of cases, while *Staphylococcus aureus* is identified in 12–47% of cases. Streptococci and Enterococci are detected in around 10% of instances, whereas gram-negative bacteria like *Escherichia coli* are observed in less than 5% of cases [4].

Conventional treatment involves local cleaning, mechanical and chemical removal of dead tissue, intravenous administration of antibiotics (DAIR), and deciding whether to retain or replace the implant based on the situation. In acute cases, efforts are made to preserve the implant, while in chronic cases, the implant is replaced. Recently, in order to accelerate healing and reduce complication rates during the perioperative period, additional materials have been developed as supplementary topical treatments. Stimulan, a fully absorbable calcium sulfate, is effective in treating both acute and persistent infections. This biocomposite has low drainage and consistent purity and can be easily mixed with liquid, powder, and antibiotics. It is available in granular form in various sizes for direct application to the infection site and can also be injected or used as a paste [5].

Negative pressure wound therapy (NPWT) has been used in clinical practice since 1990 [3]. This method involves applying a negative pressure, followed by immediate sealing with adhesive films to create a vacuum seal. The key benefits of this therapy include reducing the wound surface, promoting granulation and formation of new tissue, effectively cleansing the wound, and continuously removing exudate. This method also prevents contamination of the wound and eliminates the discomfort caused by traditional dressings. A clean wound is essential for the success of negative pressure therapy, requiring rigorous primary surgical debridement [6].

In the last 30 years, the method has increasingly become the preferred choice for various medical applications, including skin graft fixation, suppurated wounds, lower limb ulcers, infections of vascular prostheses and parietal prostheses, and diabetic foot. It is also considered feasible for treating soft tissue infections, temporarily closing open abdomen wounds, treating tendons, implants, exposed bones, rectus anastomosis fistulae, and necrotizing fasciitis. Additionally, it shows promise for treating burns, eschar, intra-abdominal and intrathoracic infections, entero-cutaneous fistulae, lymph-cutaneous fistulae, cold sores, and MRSA-contaminated wounds [7].

Still today, there are no specific guidelines that outline indications for the use of negative pressure therapy in PJI. The same applies to biocomposite, a relatively new addition to orthopedic treatments. The use of calcium sulfate has been on the rise, being utilized as a bone void filler and for delivering antibiotics in various medical procedures such as arthroplasty, chronic osteomyelitis, open fractures, and combat wounds. With this increased usage, there has been a better understanding of both the benefits and potential complications associated with it. A few cases of wound unloading, heterotopic ossifications, and hypercalcemia have been documented, with the majority of them being asymptomatic. It has been observed that complications tend to increase when larger volumes of CS are used, particularly in subcutaneous structures and in patients at high risk [8,9].

We would like to highlight that currently there are no established orthopedic guidelines that encompass the techniques used in recent times. These techniques are currently considered as options and are not standardized. To illustrate this, we will present two intricate cases involving patients of different ages and underlying conditions. Initially, these cases were managed using traditional techniques, but later on, it was decided to utilize either NTPW or antibiotic-loaded biocomposite. Throughout the treatment process, there were multiple hospitalizations, significant additional expenses, and a decline in the quality of life for the patients.

## 2. Cases

### 2.1. CASE 1

A 46-year-old woman was admitted to the Orthopedics and Traumatology Clinic at Galati Hospital. Her medical history indicated a congenital hip dysplasia leading to secondary coxarthrosis, in an advanced stage, resulting in functional impairment and an inability to stand for extended periods. Consequently, a decision was made to proceed with total hip arthroplasty.

Upon admission, laboratory tests revealed the following results: WBC count of 10.40 × 10^3^/mm^3^ (normal range: 4–10 × 10^3^/mm^3^), neutrophil count of 7.30 × 10^3^/μL (normal range: 1.5–7/μL), and blood glucose level of 106 mg/dL (normal range: 75–105 mg/dL).

The patient underwent surgery two days later. The surgery team used an uncemented prosthesis (ATLAS). The postoperative recovery was favorable, the patient was afebrile and the surgical dressing remained clean 48 h after the surgery. She remained in the hospital for 13 days and began physical rehabilitation therapy during this period. She was able to walk with a frame, her sutures were removed, and she was discharged without any complications. She attended all recommended follow-up appointments and showed a satisfactory recovery.

**Figure 1:**
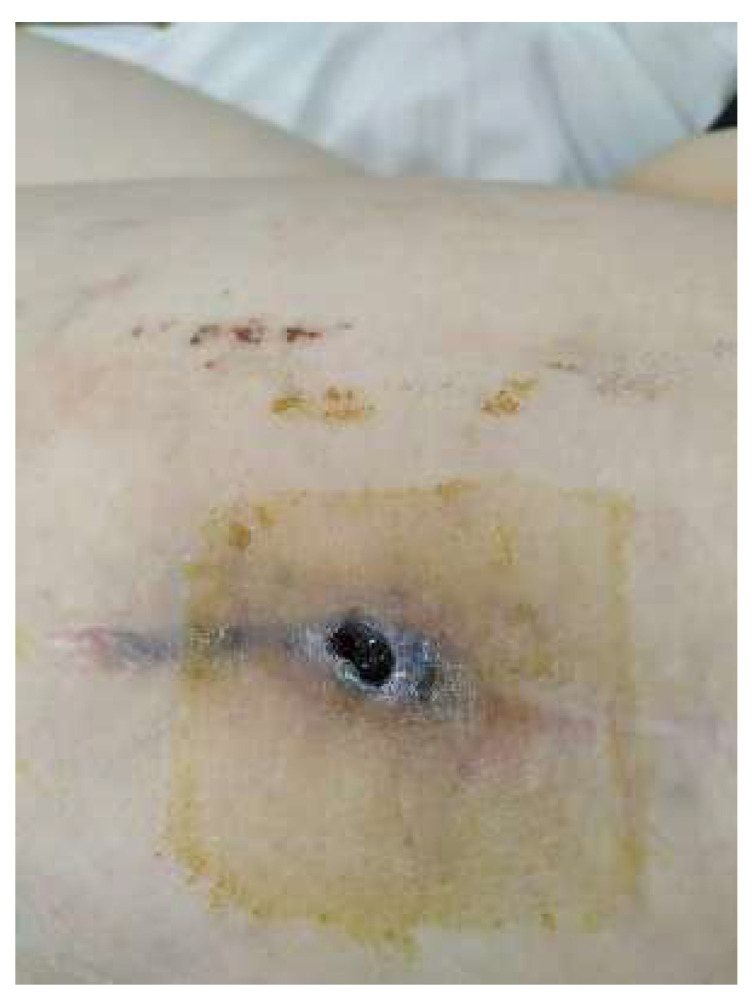
Aspect of the wound upon admission—tegumentary defect in the middle one-third of the scar with an intermittent fistula aspect and modified periregional tissue.

Four years after the initial surgery, she arrived at the emergency department complaining of pain in the operated hip, swelling around the keloid scar, and intermittent fistula in the middle third of the wound, symptoms that started 2 weeks before presentation (Figure 1).

The only lab tests that changed were the blood glucose (130 mg/dL) and fibrinogen (849 mg/dL, normal range 200–400 mg/dL), which is why we thought it was a granuloma of the deep thread. The X-ray showed no pathological aspect. The fistula was flushed daily chemically and mechanically. Local evolution was favorable with secondary closure of the fistula. The patient remained afebrile throughout hospitalization.

**Figure 2:**
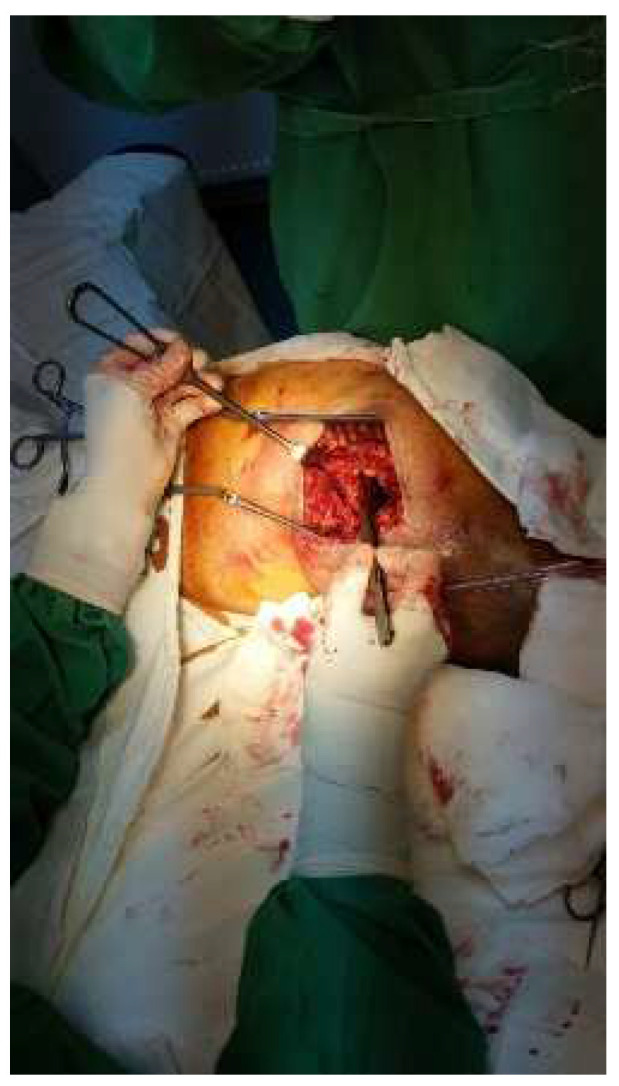
Intraoperative aspect—exposure of the left hip joint with debridement of the affected tissues.

**Figure 3:**
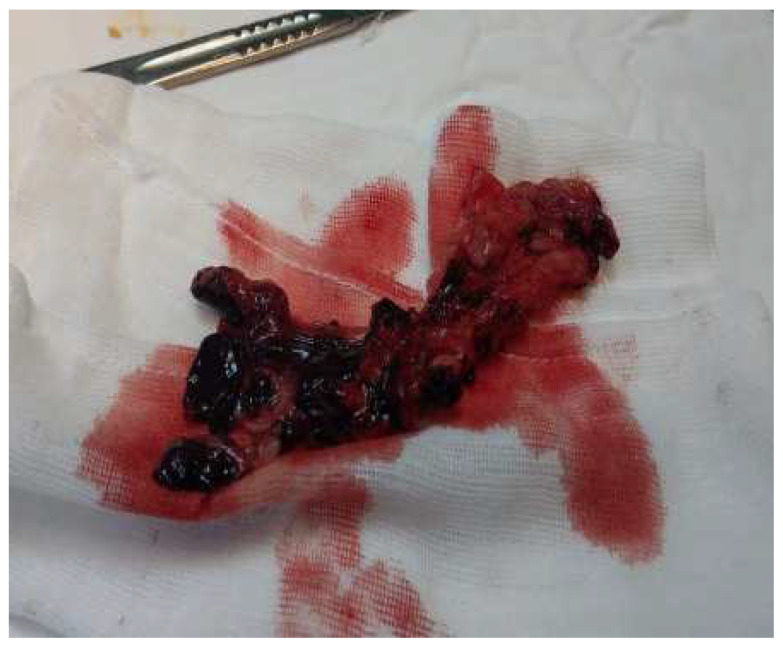
Fragments of removed tissue–modified appearance of consistency and color (metallosis).

Approximately one year after the last presentation, she returned to the emergency department with the same symptoms. Local samples were taken, and broad-spectrum antibiotic treatment was initiated. The inflammatory profile showed WBC—10.82 × 10^3^/mm^3^, fibrinogen—500 mg/dL, and ESR 45 mm/h, which is why it was decided to reintervene. We discovered several black plaques sticking to the tissue around the fistula, along with numerous thread-like pearls, which we removed (Figure 2 and Figure 3). We meticulously cleaned and disinfected the area and then closed the fistula using absorbable stitches. The patient’s recovery was positive, with a reduction in inflammation and discharge six days after the operation. A follow-up visit 12–14 days later included the removal of the stitches.

In the following year, the patient presented two more times for the same symptoms, the management and evolution being similar to the previous interventions.

**Figure 4:**
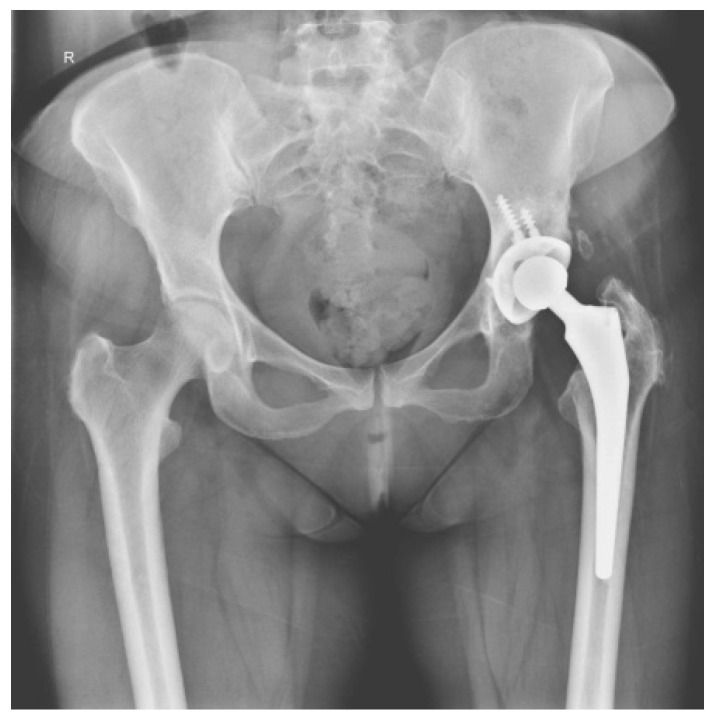
Pelvis X-ray with both joints—left total hip arthroplasty with augmented total non-cemented prosthesis with screws in the acetabulum—preoperative aspect.

**Figure 5:**
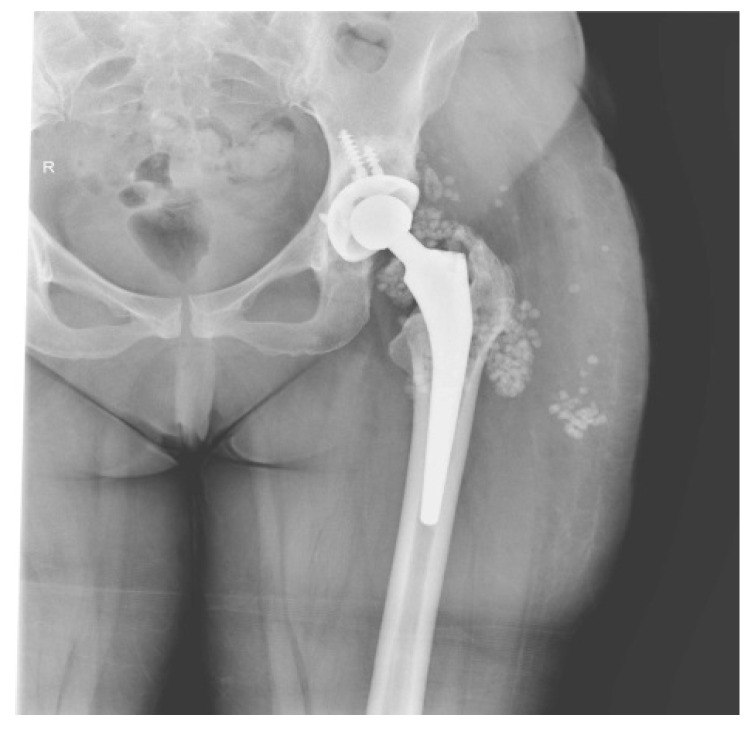
X-ray of the left pelvis—highlighting of Stimulan pearls around the prosthesis chamber and in the layer adjacent to the skin.

Six months later she came back. The pelvis X-ray showed no changes (Figure 4). It was decided to re-intervene up to the prosthesis room, where numerous areas of metallosis were evidenced, which were removed. In view of the numerous relapses and the changes discovered in the present hospitalization, we decided to apply the biocomposite in addition to the classical treatment (calcium sulfate mixed with vancomycin and gentamycin). The control X-ray after surgery is presented in Figure 5.

**Figure 6:**
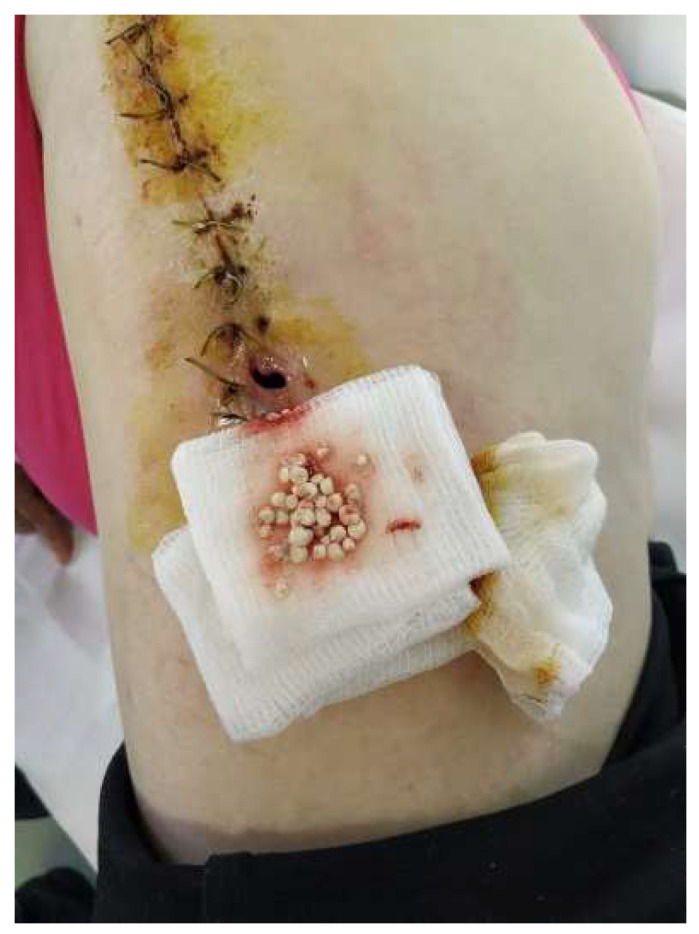
Ten days postoperative—wound dehiscence is observed at the scar site in the lower third, with about a quarter of the added pearls being externalized.

**Figure 7:**
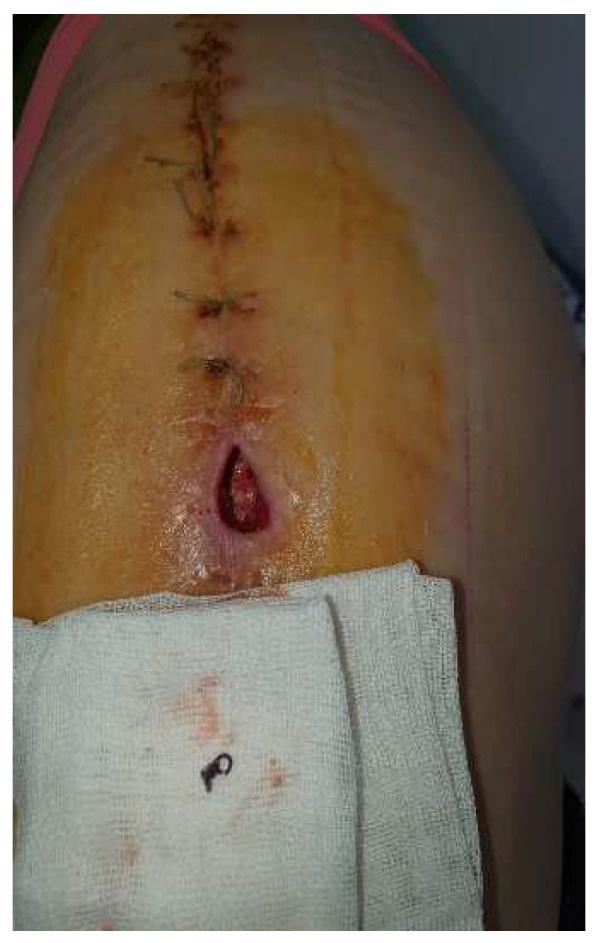
The local appearance of the wound after removal of all externalized pearls and daily grooming of the wound (red granulating wound margins).

The recovery after the surgery went well, so she was released from the hospital after 7 days. However, around 3 days after leaving the hospital, a significant amount of clear fluid and some pearls were removed from the lower one-third of the scar (Figure 6). We cleaned the area every day and removed any exposed pearls (Figure 7).

The decision was made to administer a 21-day negative pressure therapy due to the ongoing evolution. Throughout this time, there was an improvement in the inflammatory markers.

**Figure 8:**
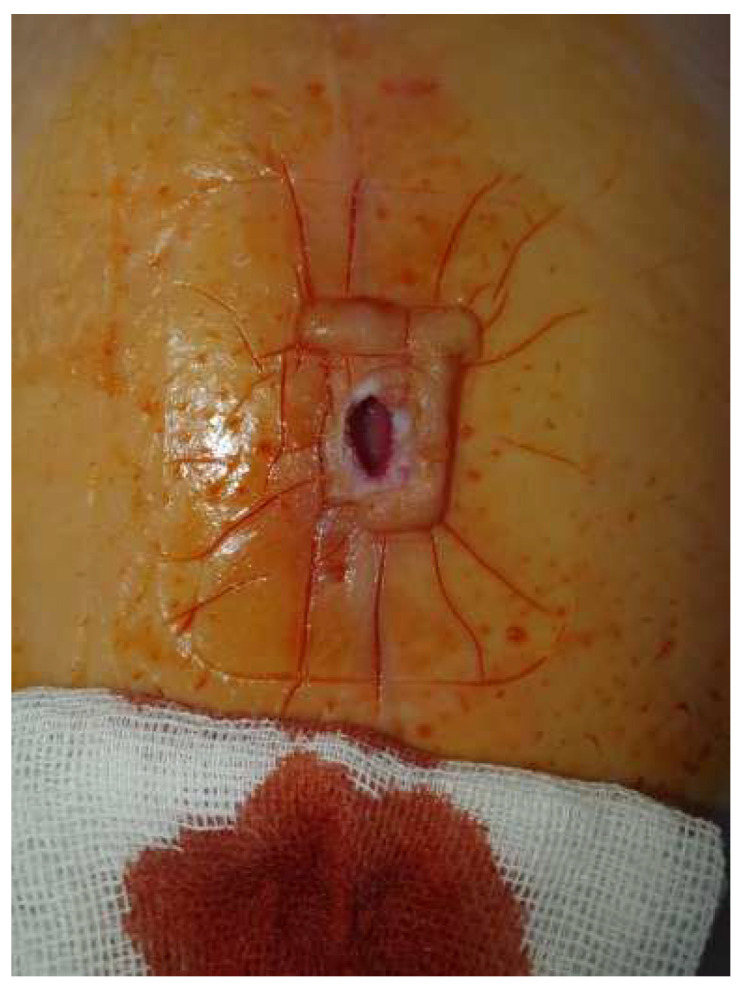
Local aspect after removing the negative pressure kit—granulation can be seen both deep in the wound and around the edges.

**Figure 9:**
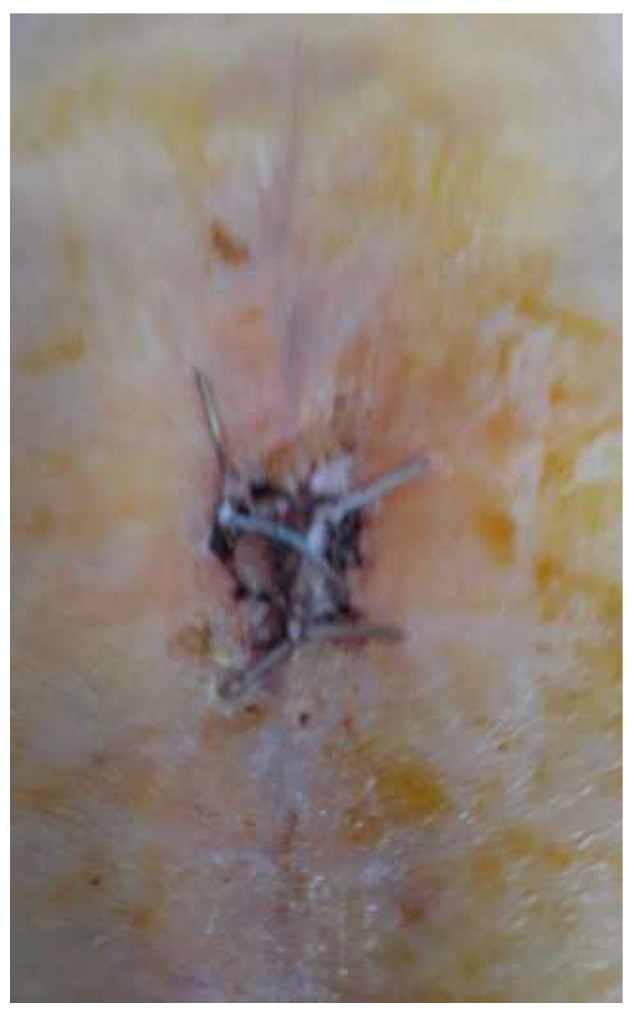
Appearance after final suture of the wound.

**Figure 10:**
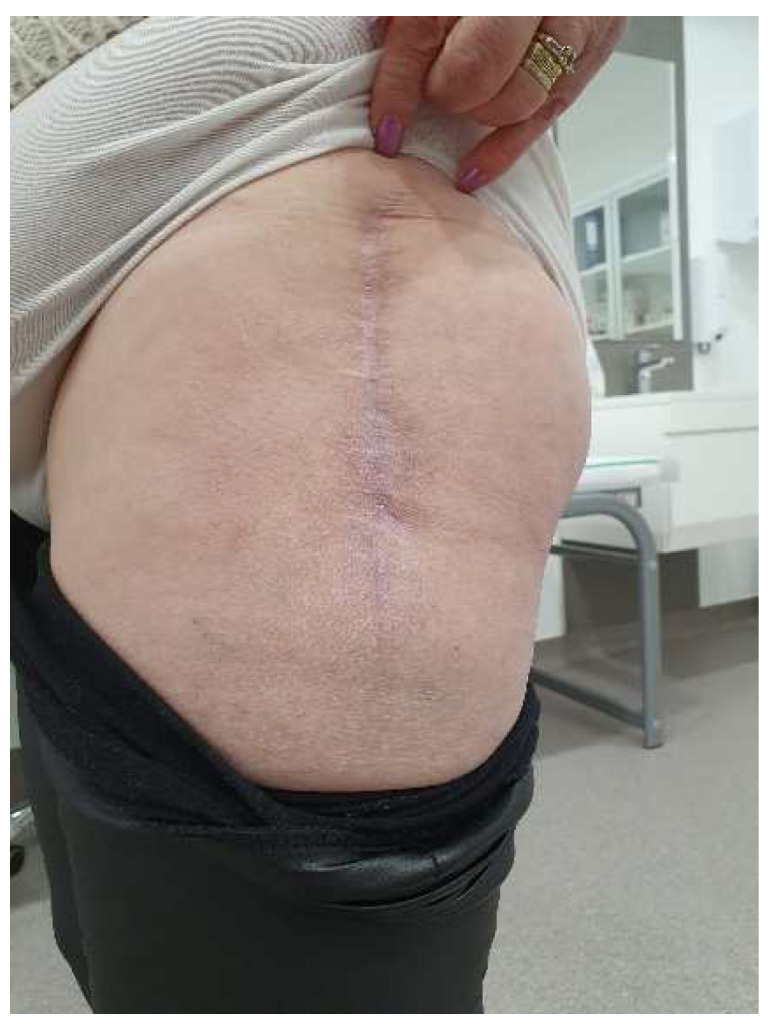
Final aspect—healed scar.

After 21 days, the lesion shrank (Figure 8), which allowed the final suture (Figure 9). The last picture (Figure 10) represents the appearance of the wound at the end of the treatment.

### 2.2. CASE 2

The second case is that of a woman who was diagnosed in 2006 with a giant cell tumor in the right femoral neck. From 2007 to 2017 she underwent 16 surgeries on her right hip.

**Figure 11:**
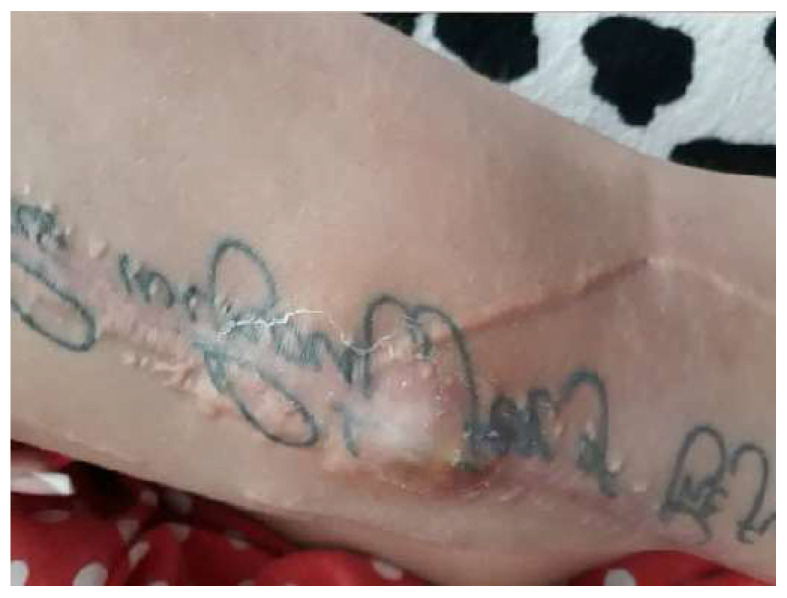
Keloid scar—in the lower third of the post-operative scar (knee region), a sub-tegumentary collection can be seen bulging the skin.

**Figure 12:**
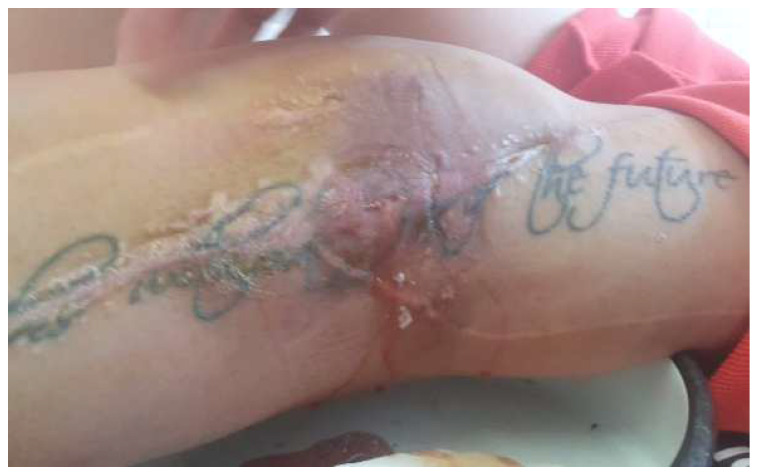
Collection starting to externalize—seropurulent secretion with wheals (about 45 mL modified content).

In 2018, she underwent surgery in Germany (Münster University Hospital) when a prosthesis was implanted in order to reconstruct the femur, acetabulum, and right knee. Following the surgery, towards the end of 2018, she developed an intermittent fistula. In 2021, she came to our clinic with this purulent collection (Figure 11 and Figure 12).

Samples were collected from the wound that tested positive for *Staphylococcus aureus*, so the patient was started on antibiotic treatment based on the antibiogram results.

Serial X-rays were performed, which showed no changes.

The patient had initially responded well to injectable antibiotic treatment and local dressing, which led to temporary wound healing and closure of the fistula. However, in 2023, the patient presented at the clinic with recurring symptoms.

Following preoperative preparation (normal inflammatory blood labs), surgery was performed during which an incision was made on the existing scar. Fistulotomy was then performed to the skin and the fistula was traced to the prosthesis chamber.

**Figure 13:**
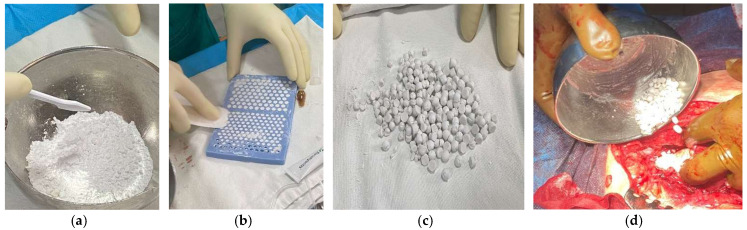
Sequence of images from the preparation of Stimulan pearls. (**a**) Mixing the biocomposite powder with Vancomycin and Gentamicin powders and blending with the liquid content until a paste is formed. (**b**) Adding the mixed paste into the silicone mold to form the pearls. (**c**) Final appearance of Stimulan pearls—different sizes. (**d**) Application of Stimulan pearls at the site of infection.

We conducted chemical and mechanical cleaning, removed damaged tissues, and performed thorough irrigation. Following thorough irrigation, Stimulan pearls were prepared and applied deeply on both sides of the prosthesis (Figure 13). Then, suturing was performed at anatomical levels.

**Figure 14:**
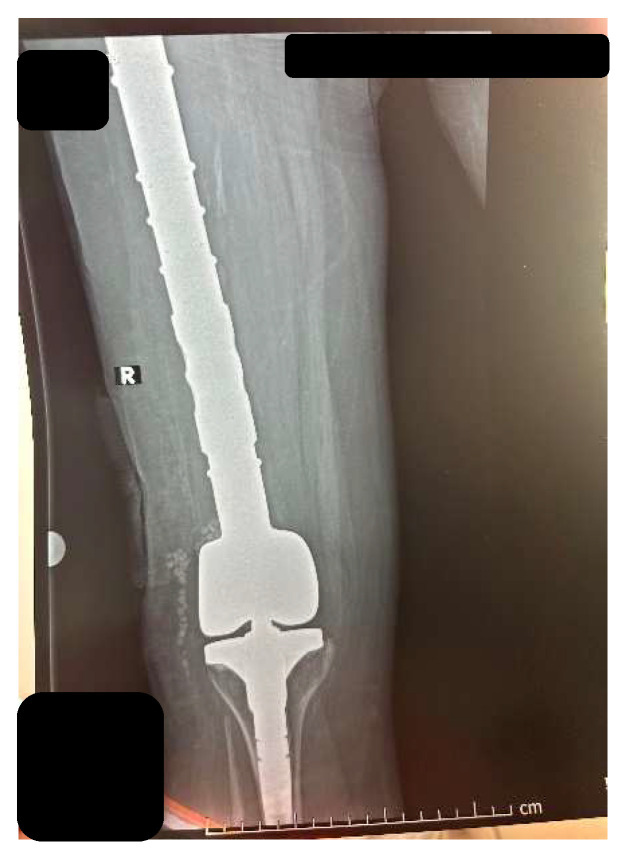
Control X-ray of the prosthesis and biocomposite—in the knee region the position of the Stimulan pearls on the side.

The local and general recovery went well, and the surgical wound had a normal appearance. The following day, we performed a follow-up X-ray to check the position of the biocomposite material (Figure 14).

**Figure 15:**
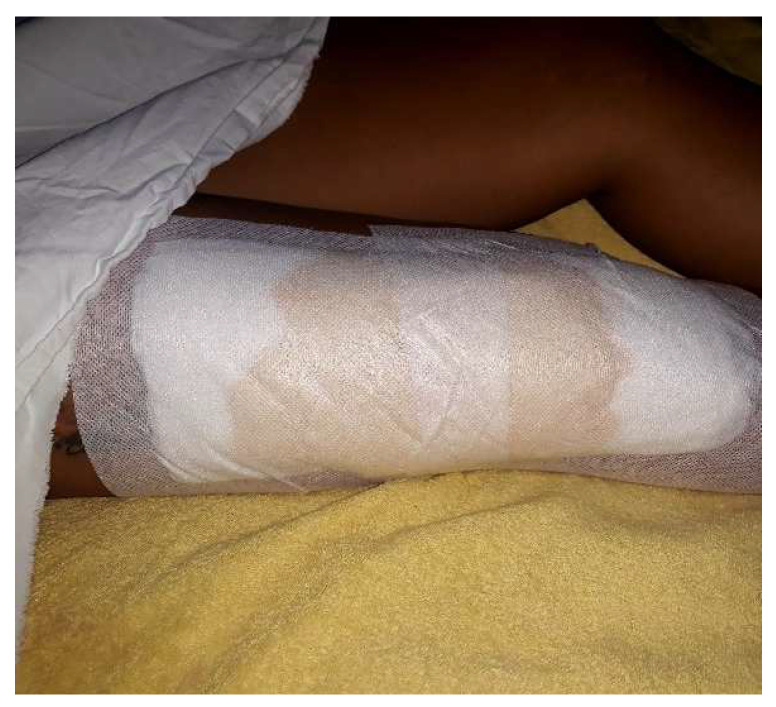
Day 5 postintervention—infiltrated dressing with serous content (about 80 mL).

On the second and third days following the surgery, there is positive progress. The bandage is easily infiltrated without any local signs of inflammation or pain (Figure 15). The bandage is replaced under sterile conditions. Antibiotic treatment is continued.

**Figure 16:**
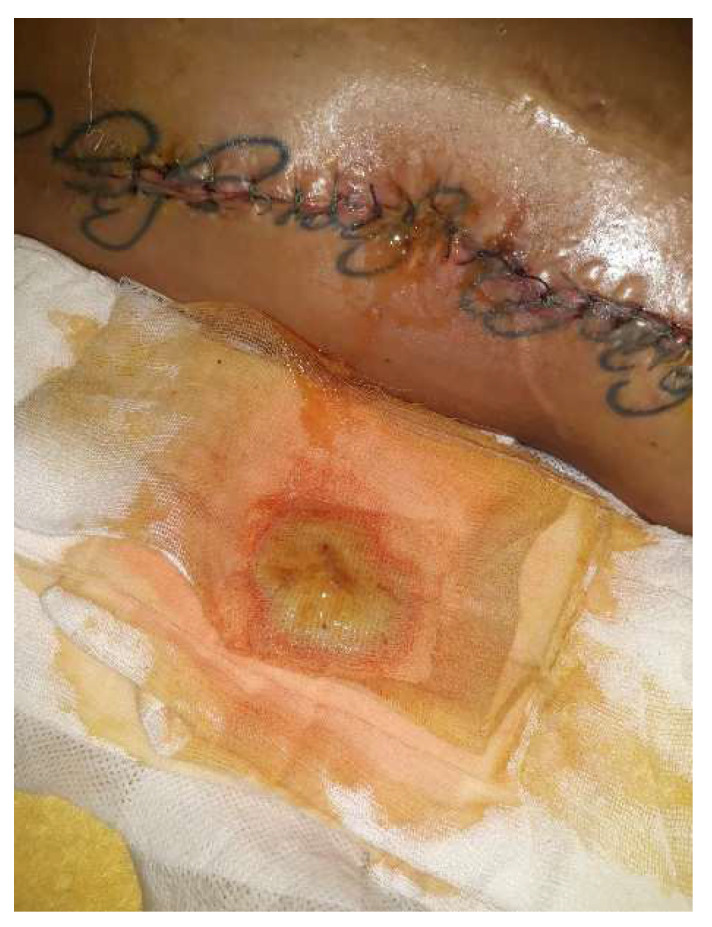
Day 5 postintervention—on the dressing, there was a yellowish seromucculent secretion and, from the wound, a continuous, serosanguininolent secretion.

**Figure 17:**
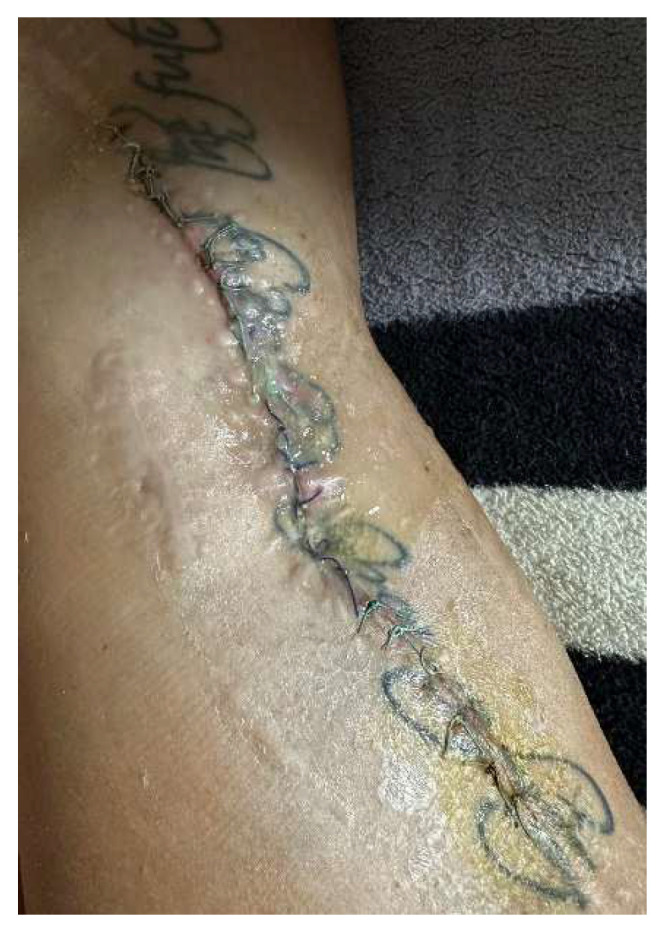
Day 10 postintervention—the same secretion but reduced in quantity, otherwise clean wound appearance.

By the fourth day, a significant amount of fluid is expelled from the wound (approximately 30 mL) (Figure 16). The antibiogram remains negative. The patient is asking to be discharged on the fifth day after the operation but comes back for the follow-up and changing of the dressing (Figure 17).

**Figure 18:**
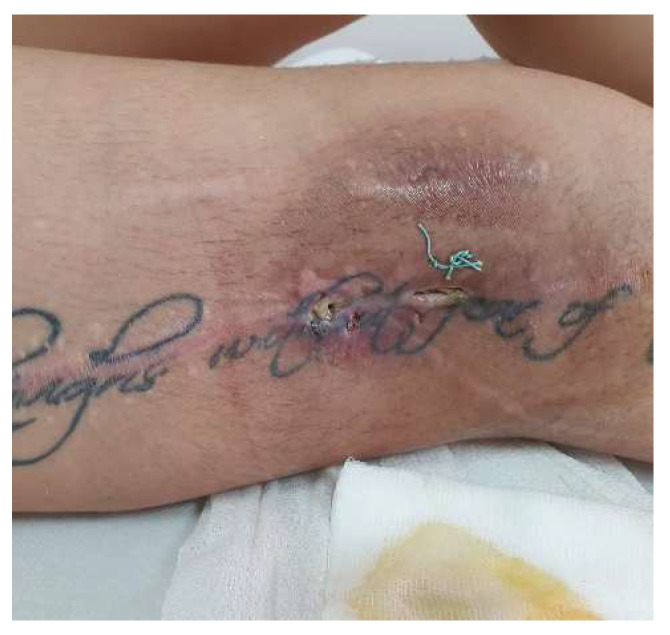
Local aspect 4 weeks after intervention—most of the sutures were removed, except in the area where the secretion was present; those sutures were subsequently removed. The local appearance is inflammatory, slightly red, with no signs of cicatrization.

By day 21, the wound showed clean healing, but there was a reddish, bulging skin lesion in the middle third of the thigh. Some sutures were taken out in a sterile environment, and a betadine dressing was applied. The patient came back for a check-up after four weeks. She was walking independently, and her thigh looked smooth. The wound had a normal keloid scar, but in the lower third of the thigh, there were two small open areas with minimal discharge (Figure 18).

**Figure 19:**
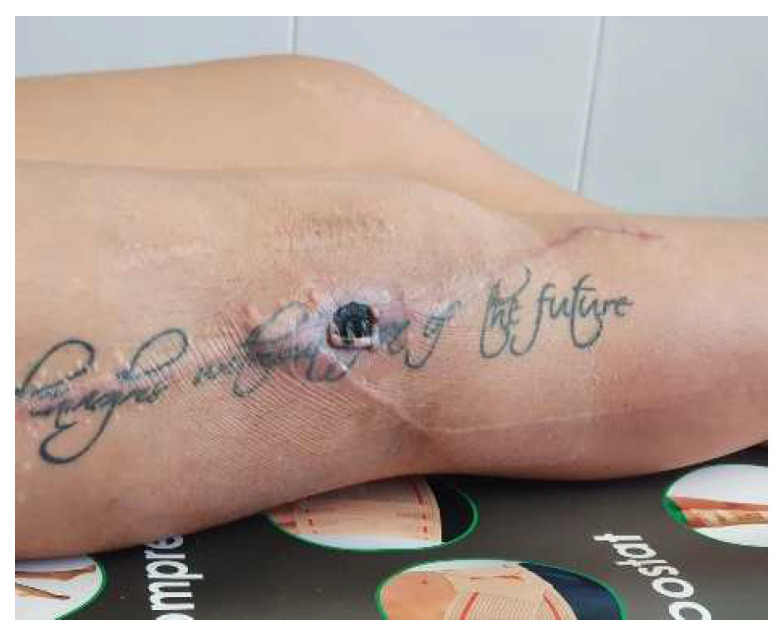
Two weeks after placing the negative pressure kit—in the area of the defect, the black sponge applied in the integumentary defect can be seen. No inflammatory signs or secondary secretion.

**Figure 20:**
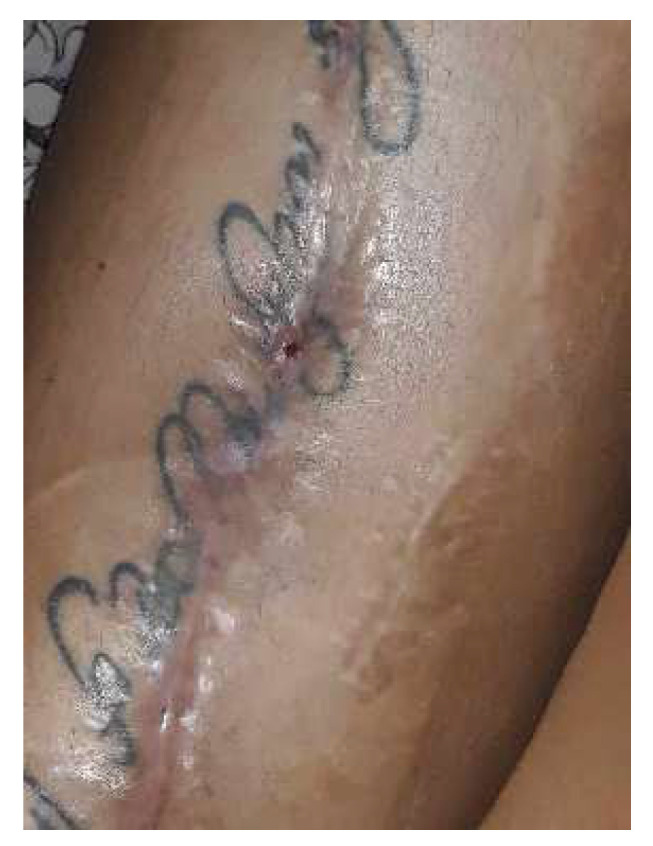
Two weeks after removing the negative pressure kit—the consistent narrowing of the skin defect. Thigh and knee were smooth without inflammatory manifestations and without secondary secretion.

The wound culture tested negative. In light of the slow progress, we opted to pursue an additional approach (NPWT) to speed up the healing process and reduce the chance of getting reinfected. Another goal was to lower the risk of having to be readmitted or undergoing further interventions. After 14 days, the wound looked clean and reddish, with the edges showing signs of granulation, so the decision was made to continue the negative pressure treatment (Figure 19). As the wound continued to heal, the negative pressure kit was eventually removed. The wound was then treated daily with a sterile dressing and betadine. Both the local and overall healing progress were positive, and the skin defect left after the removal of the sponge healed on its own (Figure 20).

## 3. Discussion

The occurrence of serious infections in deep surgical wounds following total joint replacement has decreased significantly due to advancements in operating room practices and surgical methods, thorough preoperative evaluation of the patient, and the preventive use of antimicrobial medications. Nevertheless, when it does happen, it presents a significant challenge.

The formation of biofilm is believed to be the main microbial factor contributing to the observed results of managing PJI. Bacteria within a mature biofilm are up to 10,000 times more resilient to antibiotic treatment compared to their freely suspended planktonic counterparts [10].

The identification of this aspect and the findings from subsequent research have suggested that effective approaches to eliminating biofilms could improve treatment outcomes, decrease complications, and reduce the need for complex procedures. Directly applying local antibiotic therapy at the infection site holds promise in achieving these objectives. Destroying biofilms may enhance the effectiveness of current treatment approaches, expand the suitability of minimally invasive surgical interventions, and, in certain instances, eliminate the need for surgery entirely.

Treating patients with PJI presents several challenges. The management of those who test positive for a particular bacterium has been established through guidelines and protocols [11]. On the other hand, dealing with patients who test negative for a PJI presents a greater challenge to surgeons and the multidisciplinary team because there is a lack of clear guidance in such cases.

Upon clinical findings and investigations pointing to the diagnosis of PJI, and with patients deemed suitable and willing to undergo surgery, they are evaluated for a DAIR, single-, or two-stage revision procedure [12].

The two cases we presented do not meet any of the mentioned criteria. In the first case, the condition followed an atypical, non-specific course with negative cultures and no change in radiological appearance. In the second case, we did manage to identify the pathogen (S. aureus), but the underlying condition for which the prosthesis was introduced was a specific one.

As we mentioned before, the severity of PJI has led to numerous studies aimed at identifying risk factors for infection. These risk factors can be categorized as intrinsic (related to the patient) or extrinsic (related to the environment). Approximately 80% of patients undergoing arthroplasty procedures have modifiable risk factors, with the most common being obesity (46%), anemia (29%), malnutrition (26%), and diabetes (20%). Other factors, found in smaller percentages, are rheumatoid arthritis and its treatments, smoking, malnutrition, anticoagulant use, poor hygiene, and dental infections. Unmodifiable risk factors include age, male gender, previous surgeries or infections, revision arthroplasty, and colonization of the urinary tract or nasal passages with S. aureus. [13,14].

Patients at high risk (several risk factors, mainly non-modifiable ones) with soft tissue abscesses become a challenge, with overall status limiting response to treatment [15].

The two cases have different risk factors. The first patient had class 1 obesity as the sole risk factor. The second patient had a history of multiple surgeries before the hip reconstruction surgery, in addition to the primary condition of a giant cell tumor at the femoral neck. Despite the differing risk factors, both cases involved a prolonged treatment course with multiple interventions.

In a study by Shallcross L. J. et al. [16], it was found that a substantial proportion of patients re-consult their physician for the recurrence of a soft tissue abscess within one year of the first diagnosis. The rate of recurrent infection was strongly associated with antibiotic treatment performed 6 months before the first consultation of the patient with soft tissue abscess.

Regarding the treatment, in both our cases, we chose to add to the standard approach a calcium biocomposite in combination with a topical antibiotic (Stimulan) during the surgery, directly applied at the infection site in order to obtain better control at the infection site. There are various options for managing dead space, such as using bone grafts, calcium sulfate, calcium phosphate substitutes, or PMMA. The latter options provide the advantage of delivering high levels of antibiotics over a prolonged period in a controlled environment, without the added risk of systemic side effects from prolonged antibiotic use. [17,18].

Metsemakers et al. [19] concluded in their review that successful treatment of bone and joint infection requires not only bony stability and soft-tissue cover but also effective debridement and irrigation, as well as the use of systemic and local antibiotics. They also suggested conducting prospective multicenter studies in order to assess the safety and potential toxicity associated with high local antibiotic levels.

In their analysis of ceramics, Ene et al. [20] determined that calcium sulfate exhibits superior degradation, mechanical, and antibiotic elution properties, positioning it as a promising component in future approaches to managing musculoskeletal infections. Our findings can be evaluated alongside other research, such as the Oxford studies conducted by Ferguson et al. [21].

Another noteworthy aspect was the externalization of Stimulan pearls post-surgery in the first case, with no other complications related to the biocomposite. In prior research, the drainage rates of wounds were examined in 250 revision arthroplasties after adding antibiotic-impregnated calcium sulfate beads. The research found a 3.2% drainage rate, which was more common in patients with higher bead volumes, subcutaneous placement, and poor overall health. [22].

Although there was a faster improvement in both our cases, the issues were not completely resolved, prompting us to implement negative pressure therapy after resolving the infection.

The system of NWPT has seen widespread uptake and is now implemented routinely for open wounds, such as open fractures, fasciotomies, ulcers, and infected wounds. Termed Vacuum-Assisted Closure (often abbreviated to “VAC”), this system is only effective if applied correctly by trained individuals. It is usually performed in the operating room, given the fact that the wounds usually require debridement and a washout in a sterile environment.

NWPT may be used in several scenarios, including the treatment of septic wounds, necrotizing fasciitis, gas gangrene, and venous or pressure ulcers. In the orthopedic fields, it can be used especially in open fractures, infections (including osteomyelitis and periprosthetic joint infections), and fasciotomy [23].

Although the quality of the available literature is mostly poor, there are several studies that support the effectiveness of negative pressure therapy in reducing the risk of infection, accelerating the wound healing process, and reducing hospitalization length, at least in open fractures [24,25].

In particular, positive outcomes in terms of hospital stay, wound size reduction, wound healing time, and deep infection rate were reported by Kumaar et al. [26] in their randomized controlled trial comparing NPWT with standard dressing in open fractures.

Incisional NPWT (INPWT) can be used “for prophylactic purposes” on several occasions, especially in high-risk patients (diabetes, glucocorticoid therapy, very elderly, revision surgery). Several studies support this kind of approach [27]. A meta-analysis conducted by Ailaney et al. [28] supported the efficacy of INPWT in revision total hip arthroplasty, decreasing hospital stay and reoperation rates.

The benefits of using biocomposite are quite obvious. Directly administering antibiotics to the infection site offers several advantages compared to giving them systemically. It allows for higher concentrations of the medication to reach the infected tissue while minimizing exposure to the rest of the body. This approach may reduce the occurrence of antibiotic-related side effects and help prevent antimicrobial resistance by preserving the natural balance of microorganisms in the gastrointestinal tract. There are limitations, however, such as high acquisition costs, making it unavailable in many orthopedic centers. In addition, there are other theoretical drawbacks. Even though local delivery results in much lower systemic antibiotic levels, there is still a risk of systemic complications such as nephrotoxicity [29]. It is important to view local antibiotic therapy as a complement to surgical treatment, including thorough debridement, dead space management, and implant exchange. Despite optimal antibiotic delivery, rising rates of antibiotic resistance may still render the therapy ineffective in the future.

Even so, the use of biocomposite is becoming more common, allowing for additional research to better understand its applications and potential complications or limitations. On the other hand, NPWT is not widely utilized in orthopedics at this time. It is primarily employed in primary arthroplasty to expedite healing, with limited studies on its use after PJI. Consequently, there are currently no established guidelines governing its usage, which represents a significant limitation. The costs associated with negative pressure dressings are another limitation, and a more detailed understanding of their possible benefits in PJI is an essential aspect that needs to be conducted in future studies before they can be used on a larger scale.

Along with PJI’s new approaches, we want to emphasize the importance of prevention. Regardless of the treatment one chooses, it is important to schedule postoperative follow-up appointments at two and six weeks, six months, one year, and annually thereafter. During these follow-up visits, clinical symptoms and signs of infection should be monitored, and tests for CRP and ESR levels should be conducted. Additionally, plain radiographs, which include an AP pelvis and lateral of both hips, should be performed at each follow-up appointment.

## 4. Conclusions

Our experience has shown that the combination of surgical debridement and the insertion of calcium sulfate beads has been successful in treating bone and joint infections without any adverse effects or complications. Stimulan is a highly adaptable polymeric material that can be combined with a wide range of antibiotics to better suit various bacteria, their antibiograms, and the requirements of surgeons. These characteristics make it a valuable supplement for dealing with complex infection cases and for their prevention. Additionally, calcium sulfate beads are successful in managing bone and joint infections without causing significant side effects or complications.

The successful negative pressure therapy, which has shown excellent outcomes in treating open or chronic wounds, has now been expanded to include arthroplasty. NPWT proved to be a beneficial tool for treating complicated wounds, as shown in the cases we presented. It creates both macro and micro changes, which can enhance wound healing and reduce the buildup of hematoma and seroma, leading to a drier wound with a lower risk of healing complications and infection.

In our cases, the addition of these techniques had other obvious benefits. They helped decrease the frequency of dressing changes, leading to a shorter hospital stay and enhanced comfort for the patient.

Additional research is necessary to determine the circumstances in which NWPT and biocomposites should be utilized from the beginning, in order to accelerate recovery, enhance patients’ quality of life, facilitate early social reintegration, and reduce the strain on healthcare resources by minimizing the need for repeated interventions. Nonetheless, it is essential to remotely monitor patients who have already been included in order to evaluate their long-term prognosis and rates of reinfection.

## Data Availability

The data published in this research are available on request from the first author and corresponding authors.
